# Supplementary figures and images for: Systems Analysis of Gut Microbiome Influence on Metabolic Disease in HIV-Positive and High-Risk Populations
Source: mSystems. 2021 May 18;6(3):e01178-20. doi: 10.1128/mSystems.01178-20 (PMC8269254; doi:10.1128/mSystems.01178-20)

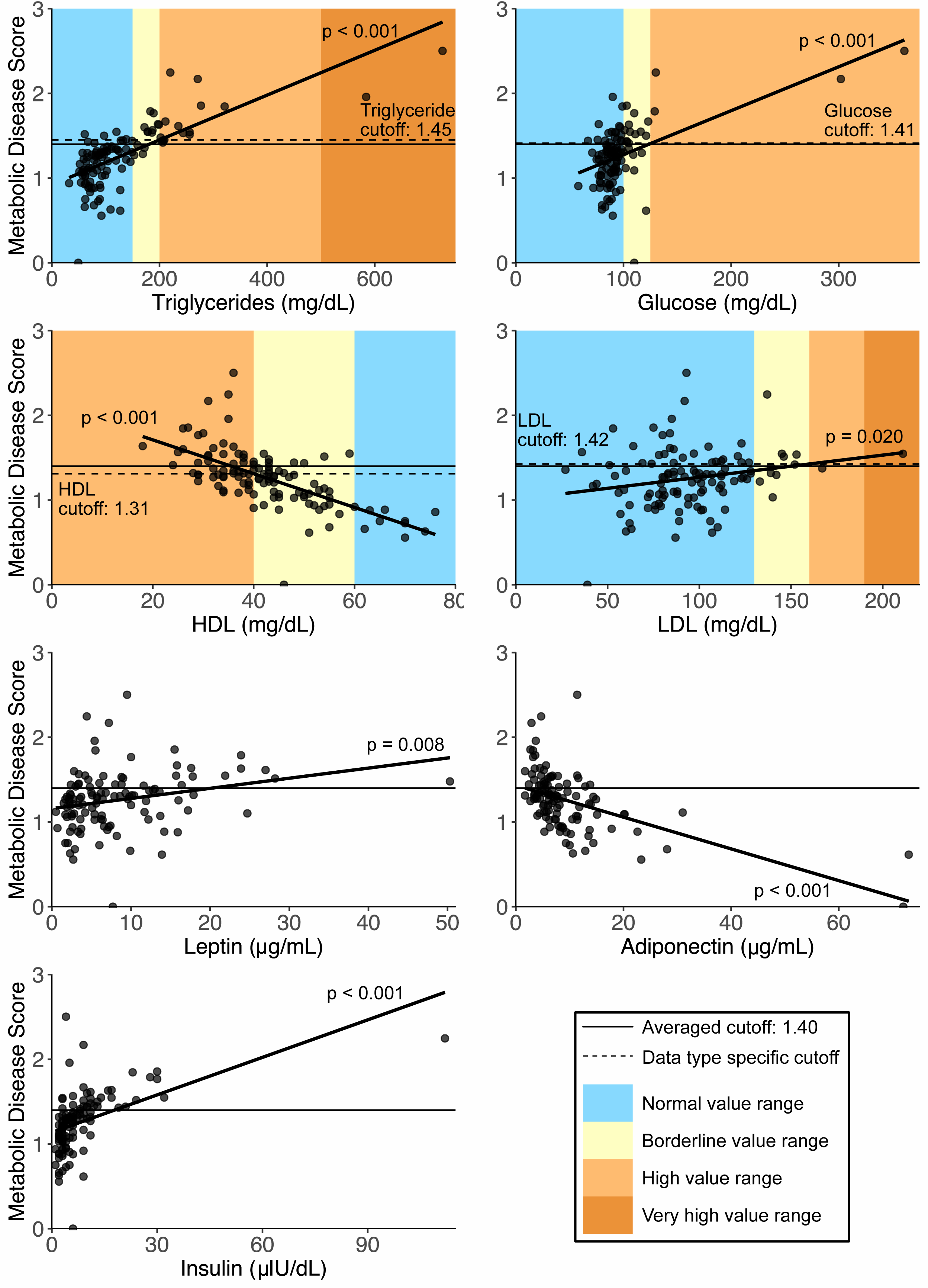

Supplement: FIG S1 [file msystems.01178-20-sf001.tif]

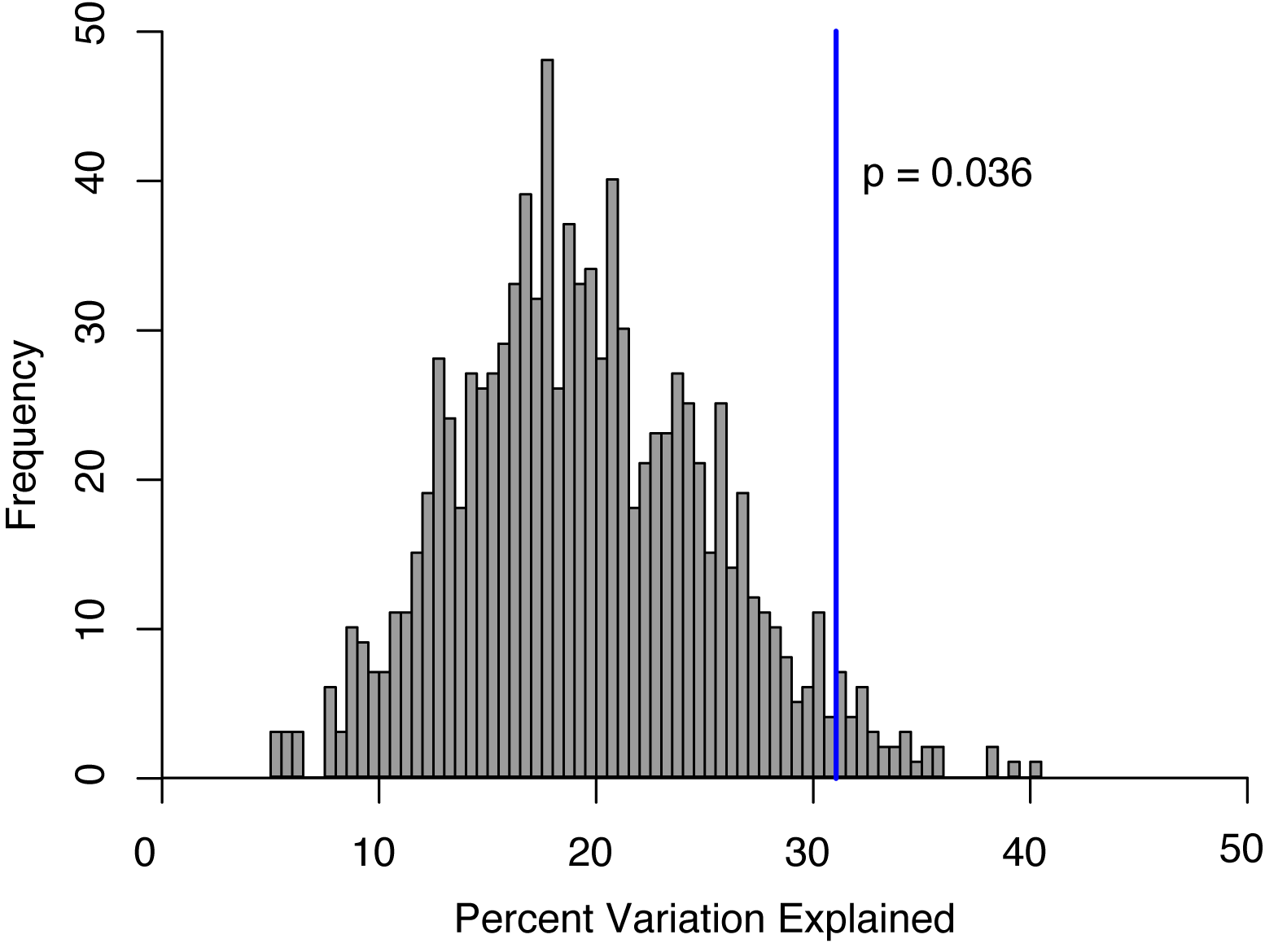

Supplement: FIG S2 [file msystems.01178-20-sf002.tif]

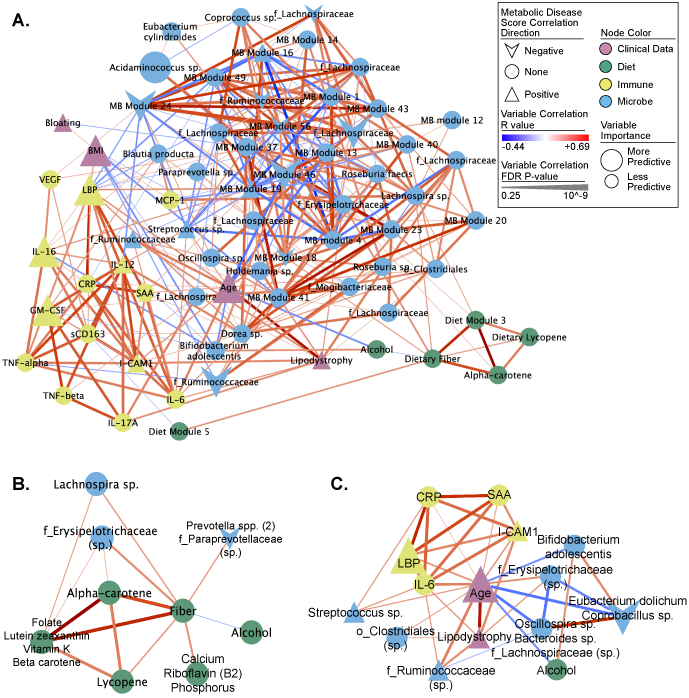

Supplement: FIG S3 [file msystems.01178-20-sf003.tif]

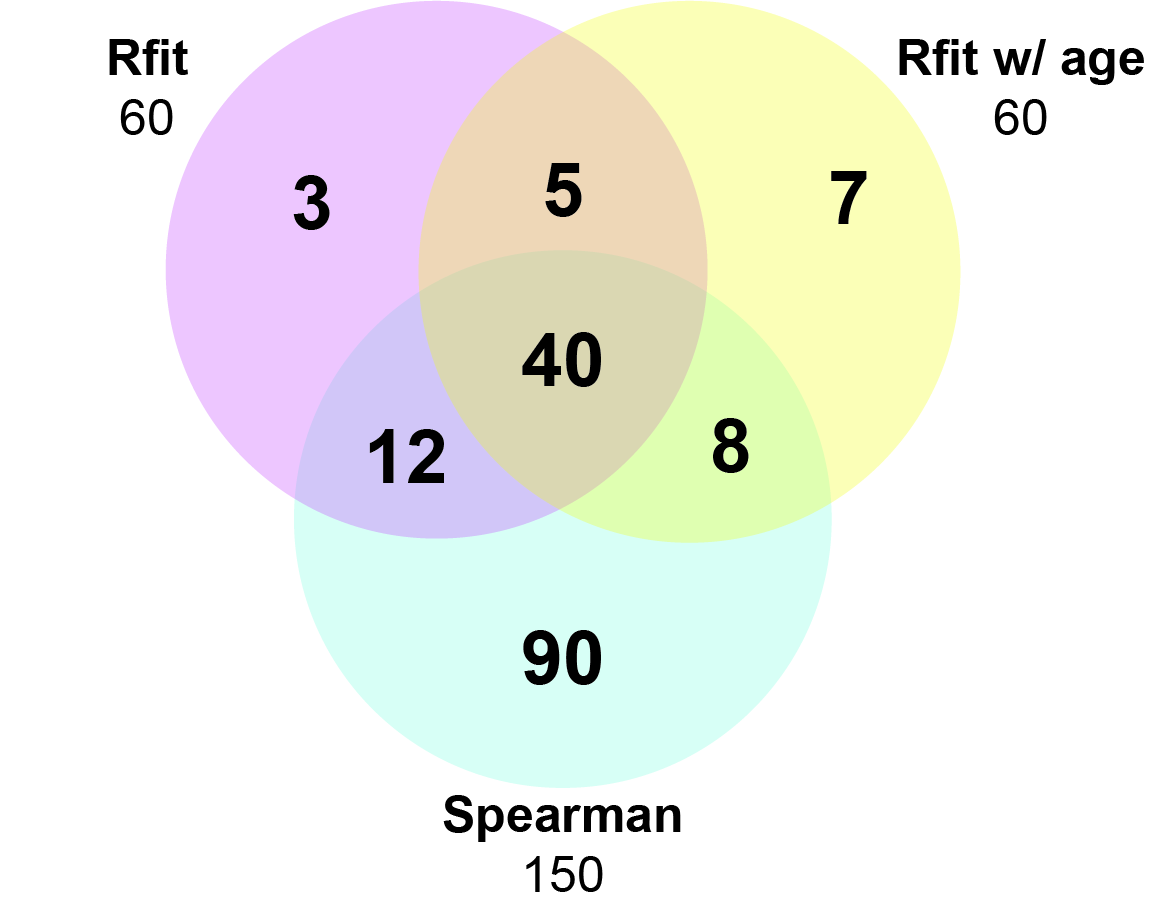

Supplement: FIG S4 [file msystems.01178-20-sf004.tif]
